# A meta-analysis of the effects of DBS on cognitive function in patients with advanced PD

**DOI:** 10.1515/med-2025-1292

**Published:** 2025-10-09

**Authors:** Yuxin Gai, Mengyi Qian, Guojian Lin, Hujie Zhan, Linhui Fan, Lijing Su

**Affiliations:** Department of Neurology, Ningbo Yinzhou No. 2 Hospital, Ningbo, 315100, Zhejiang, China; Department of Neurosurgery, The First Affiliated Hospital, Zhejiang University School of Medicine, Hangzhou, Zhejiang, China; School of Medicine, Shaoxing University, Shaoxing, Zhejiang, China; College of Pharmaceutical Science, Zhejiang University of Technology, Hangzhou, Zhejiang, China; The Second Clinical Medical College, Zhejiang Chinese Medical University, Hangzhou, Zhejiang, China; Department of Neurology, Ningbo Yinzhou No. 2 Hospital, 998 North Qianhe Road, Yinzhou District, Ningbo, 315100, Zhejiang, China

**Keywords:** deep brain stimulation, Parkinson’s disease, cognitive function, language function, meta-analysis

## Abstract

**Background:**

Although deep brain stimulation (DBS) has been proven to enhance motor function in Parkinson’s disease (PD) patients, its potential adverse impact on cognitive function remains ambiguous. This study aimed to explore the effects of DBS on cognitive function in patients with advanced PD.

**Methods:**

PubMed, EBSCO, Cochrane Library, Web of Science, and Embase were searched for randomized controlled trials (RCTs) and cohort studies on DBS and advanced PD from inception to January 2025. The main cognitive function assessment tools include but are not limited to Mini-MENTAL State Examination (MMSE) and Mattis Dementia Rating Scale (MDRS).

**Results:**

A total of 7 RCTs and 11 cohort studies were included. Analyses reveals no significant differences in MMSE (mean difference [MD] = −0.33, *P* = 0.19) and MDRS (MD = −0.75, *P* = 0.08) between the DBS group and the best medical therapy (BMT) group overall. However, the DBS group had significantly worse cognitive function after treatment than the BMT group in phonemic fluency (MD = −3.17, *P* = 0.03). No significant differences were observed between the groups in other domains, including information processing, memory, executive function, and visuospatial function.

**Conclusions:**

DBS poses a potential risk to cognitive function in patients with advanced PD.

## 1 Introduction

Parkinson’s disease (PD) is the second most common neurodegenerative disease worldwide, predominantly affecting middle-aged and older adults. The global prevalence has risen steadily. In 2019, PD caused 5.8 million people to be disabled worldwide, representing an 81% increase from 2000 [1,2]. Characterized by three primary motor symptoms – rigidity, resting tremor, and bradykinesia – PD significantly impairs patients’ quality of life.

There is growing evidence that pharmacological treatments are ineffective for patients with advanced PD [3]. In recent years, treatments that use technology to affect specific locations in the brain have become increasingly common. One of the most important and accepted treatments is deep brain stimulation (DBS) [4]. Limousin and Foltynie [5] analyzed the efficacy of DBS in the treatment of PD, concluding that DBS improves motor function for up to 10 years, and that the prevalence and severity of dementia among patients receiving DBS is comparable to that among those treated with best medical therapy (BMT). Their study confirmed the effect of DBS on motor deficits, but for cognitive function, the efficacy was questionable. The results of a retrospective study by Hong et al. [6] reported no significant difference in overall cognition in patients with PD compared to preoperative (Mini-MENTAL State Examination [MMSE]: *P* = 0.275). However, Acera et al. [7] reported a 5-year follow-up of 40 patients with PD and found that the patients’ semantic and phonological fluency scores decreased by 16.1 and 16.6%, respectively. That is, the patients’ cognitive function declined in terms of language. In addition, due to pathological reasons or aging factors, patients with PD have symptoms of reduced cognitive function themselves. Based on the current studies, it remains inconclusive whether DBS affects cognitive function in patients with advanced PD.

DBS (specifically stimulation of the subthalamic nucleus [STN] and globus pallidus [GPi]) was first approved by the Food and drug administration for the treatment of PD in 2003 [8]. Many newer studies have been reported recently. Therefore, the aim of this study was to explore the changes in cognitive function after DBS in patients with advanced PD compared to the BMT.

## 2 Methods

### 2.1 Search strategy

This meta-analysis adheres to the Preferred Reporting Items for Systematic Reviews and Meta-Analyses, this meta-analysis was registered with the international prospective register of systematic reviews under the registration number CRD420250633442. We conducted comprehensive searches from their inception through the databases till January 2025 across five electronic databases: PubMed, EBSCO, Cochrane Library, Web of Science, and Embase. The searches aimed to explore the effect of DBS on cognitive function in patients with PD. To minimize potential omissions, references from the included studies were manually searched. Search terms were developed using a combination of Medical Subject Headings (MeSH) and free-text terms as follows: Parkinson Disease [MeSH] OR PD OR Parkinson’s disease OR parkinsonian disorder OR parkinsonism disease OR Parkinson diseases OR Parkinson’ disease OR parkinsonian disease OR parkinsonian diseases AND deep brain stimulation [MeSH] OR deep-brain stimulation OR deep brain stimulation OR deep brain electrical stimulation OR electrical stimulation treatment.

### 2.2 Selection criteria

Inclusion criteria included studies that met all of the following criteria: (1) adult patients diagnosed with advanced PD; (2) the experimental group received DBS combined with BMT whereas the control group received only BMT (dopamine-based medication); (3) reported cognitive functioning scales included, but were not limited to MMSE, Mattis Dementia Rating Scale (MDRS), and phonemic fluency; and (4) study designs were randomized controlled trials (RCTs) or cohort studies.

Exclusion criteria included studies if they met any of the following: (1) non-English language publications, (2) lack of availability of the full text, (3) absence of statistically available data; and (4) studies that were superseded by the most comprehensive or more recent publications when the trail was updated.

### 2.3 Outcome measures

PD is a neurological disease caused by a progressive decrease in nigrostriatal dopamine in the brain, also known as tremor paralysis. Internationally, in order to assess the severity of PD, it is classified into five stages based on the Hoehn-Yahr classification. Stage 0: asymptomatic. Stage 1: unilateral body is affected but not balance. Stage 2: body is affected bilaterally but not balance. Stage 3: balance is affected with mild to moderate clinical symptoms but the patient is able to live independently. Stage 4: mobility is severely impaired, but patients can walk and stand on their own. Stage 5: confined to bed or a wheelchair without help from others. DBS is not currently recommended for patients with early-stage PD in the guidelines [8], which is still in the clinical research stage. In this study, patients with advanced PD are defined as those with stage 3 or higher disease who require DBS.

DBS is a neurosurgical treatment that delivers continuous electrical stimulation through implanted electrodes in specific areas of the brain to modulate abnormal nerve activity. The DBS used in the study placed electrodes in the brain’s inner core, including the STN, GPi, and ventralis intermedius (VIM).

Specific scales measured included but not limited to the MMSE, MDRS, and phonemic fluency. The MMSE refers to the Brief Mental State Examination scale, which is a commonly used neuropsychological screening tool to assess an individual’s cognitive functioning, including intellectual status and the degree of cognitive decline. Higher MMSE scores indicate better cognitive function. If mean difference (MD) < 0, the statistical results tend to support the BMT group. The MDRS is primarily used to assess the presence and severity of dementia in an individual, as well as multiple indicators, such as attention and memory. Lower MDRS scores indicate better cognitive functioning. If MD < 0, the statistical results tend to favor the BMT group. Phonemic fluency is used to assess verbal fluency, a scale that measures an individual’s ability to correctly produce words beginning with specific letters or phonemes in a short period of time. Lower phonemic fluency scores indicate better language functioning. If MD < 0, the statistics tend to favor the BMT group.

### 2.4 Data extraction

Data extraction was performed independently by two researchers based on the inclusion criteria, utilizing a predefined checklist. In the event of discrepancies, the data were reviewed, and consensus was achieved through discussion. Extracted information encompassed study characteristics (authors, year of publication, country, sample size, treatment regimen, study design), patient demographics (age, gender, duration of disease), and cognitive function scales (MMSE, MDRS, and phonemic fluency, among others).

### 2.5 Quality assessment

Quality assessment was independently conducted by two researchers. RCTs were evaluated using the Cochrane Risk of Bias Assessment Tool, which includes criteria such as random sequence generation, allocation concealment, and blinding of participants, personnel, and outcome assessors. Cohort studies were assessed using the Newcastle-Ottawa Quality Assessment Scale, which includes three dimensions: selection, comparability, and outcomes, and has a maximum score of 9. In this study, studies with scores of 6 or higher were considered to be high quality.

### 2.6 Statistical analysis

Statistical analyses were performed using RevMan 5.3.0 (Cochrane Collaboration Review Manager). Dichotomous variables were assessed using MD and 95% confidence intervals (95% CI). Heterogeneity among studies was analyzed using the *I*² statistics, with *I*² < 25% indicating no heterogeneity, *I*² in the range of 25–50% indicating low heterogeneity, *I*² in the range of 50–75% indicating moderate heterogeneity, and *I*² > 75% indicating high heterogeneity. Random effect model was applied to studies with *I*² > 50%, while fixed effect model was applied to studies with *I*² ≤ 50%. Subgroup analyses were conducted based on the type of scales. All tests were two-sided, and *P* < 0.10 was considered potentially statistically significant [9] while *P* < 0.05 was considered statistically significant. If more than ten articles were included, publication bias was performed using the Begg’s test, and sensitivity was tested by excluding each study individually.

## 3 Results

### 3.1 Description of studies included

**Figure 1:**
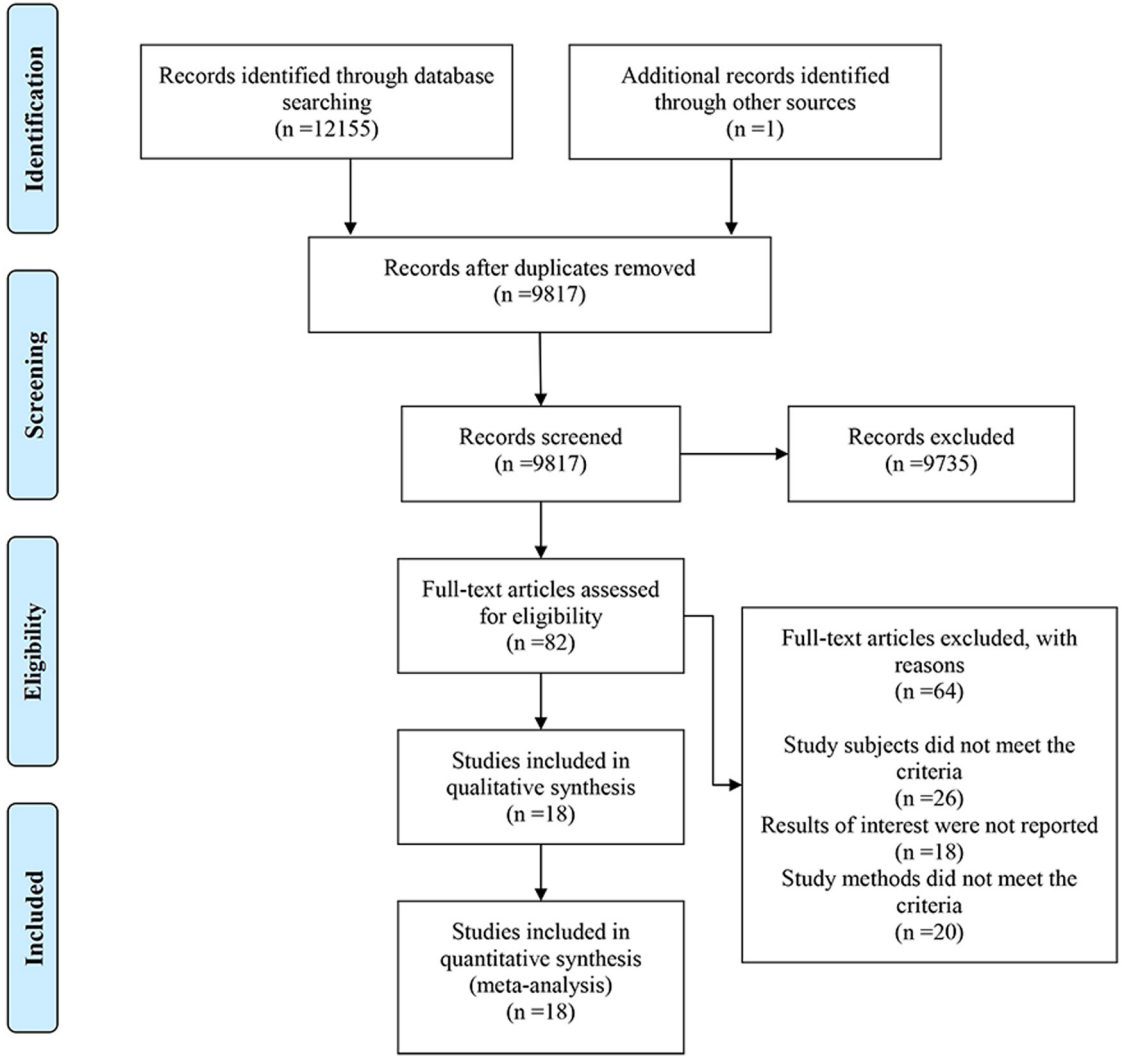
Flow chart of study selection.

A comprehensive search of five electronic databases yielded 12,155 search terms. One additional term was obtained from other sources. After removing duplicates, 9,817 studies remained. Subsequent screening of titles and abstracts led to the exclusion of 9,735 studies due to non-compliance with the study criteria. Of the 82 studies considered further, 26 were excluded due to ineligible research subjects, 18 for not reporting relevant outcomes, and 20 due to the ineligible research methods. Ultimately, 18 studies fulfilled the inclusion criteria and were analyzed [10–27] (Figure 1).

### 3.2 Characteristics of patients and trials

The 18 studies, published between 2002 and 2023, involved 974 participants. The DBS assessed included STN, Gpi, and VIM, while the BMT consisted mainly of levodopa-based treatments. Seven of the studies were conducted in the US, four in Italy, two in the UK, two in Germany, one in Denmark, one in the Netherlands, and one in Chicago. Study designs included 7 RCTs, 11 cohort studies. Detailed characteristics of these studies are presented in Tables 1 and S1.

**Table 1:** Characteristics of all the studies included in the meta-analysis

Author	Year	Country	Number		Cognitive functioning scales	Follow up (months)	Study design
			Experimental	Control			
Helle Just	2002	Denmark	11	13	PDQ-39	6	Prospective cohort study
H.M.M. Smeding	2006	Netherlands	99	36	RAVLT, Trial A, Stroop, category fluency, Trail B	6	Prospective cohort study
Roberto Cilia	2007	Italy	20	12	MMSE, category fluency, phonemic fluency	12	Prospective cohort study
Karsten Witt	2008	Germany	60	63	MDRS, phonemic fluency, semantic fluency	6	RCT
M.K. York	2008	USA	23	27	MMSE, MDRS, RAVLT, BVMT, Trial A, digit span, Stroop, Boston naming, letter fluency, Trail B	6	Prospective cohort study
Frances M. Weaver	2009	Chicago	121	134	MDRS, BVMT, Boston naming, category fluency, phonemic fluency	6	RCT
Laura B. Zahodne	2009	USA	22	19	Boston naming, category fluency	12	Retrospective cohort study
Roberta Zangaglia	2009	Italy	32	33	MMSE, digit span	36	Prospective cohort study
Ania Mikos	2010	USA	24	19	Trial A, Stroop, Trail B, JLO	16	Prospective cohort study
Adrian Williams	2010	UK	162	153	PDQ-39	12	RCT
Aristide Merola	2011	Italy	20	20	Digit span, phonemic fluency, Trail B	15	Retrospective cohort study
Amy E. Williams	2011	USA	19	18	MMSE, MDRS, RAVLT, BVMT, Trial A, digit span, Stroop, Boston naming, letter fluency, semantic fluency, Trail B	24	Prospective cohort study
Karsten Witt	2013	Germany	31	31	MDRS, letter fluency, semantic fluency	6	RCT
Aristide Merola	2014	Italy	19	16	Category fluency, phonemic fluency, trail B	/	Retrospective cohort study
David Charles	2014	USA	15	15	PDQ-39	24	RCT
Michael G. Tramontana	2015	USA	15	15	Digit span, Stroop, Boston naming, phonemic fluency, semantic fluency, JLO	24	RCT
Patric Blomstedt	2018	UK	9	10	PDQ-39	6	Prospective cohort study
Mallory L. Hacker	2023	USA	14	14	Digit span, Stroop, Boston naming, phonemic fluency, semantic fluency, JLO	60	RCT
Mallory L. Hacker	2023	USA	14	14	Digit span, Stroop, Boston naming, phonemic fluency, semantic fluency, JLO	60	RCT

RCT, randomized control trial; PDQ-39, Parkinson’s disease questionnaire-39; RAVLT, Rey Auditory Verbal Learning Test; MMSE, Mini-MENTAL State Examination; MDRS, Mattis Dementia Rating Scale; BVMT, Brief Visuospatial Memory; JLO, judgment of line orientation.

### 3.3 Quality assessment

Among the seven included RCTs, three were classified as “high risk” in selection bias due to small sample size and one was classified as “high risk” in attrition bias because of a few scale outcomes. However, all RCTs demonstrated “low risk” concerning allocation concealment, blinding of participants and personnel, blinding of outcome assessment, selective reporting of research results, and other bias (Figures S1 and S2). Among the 11 cohort studies, points were subtracted for issues such as insufficient comparability, inadequate outcome metrics, or insufficient length of follow-up. Finally, all studies scored 6 or higher (Table S2).

### 3.4 DBS effects on general ability

**Figure 2:**
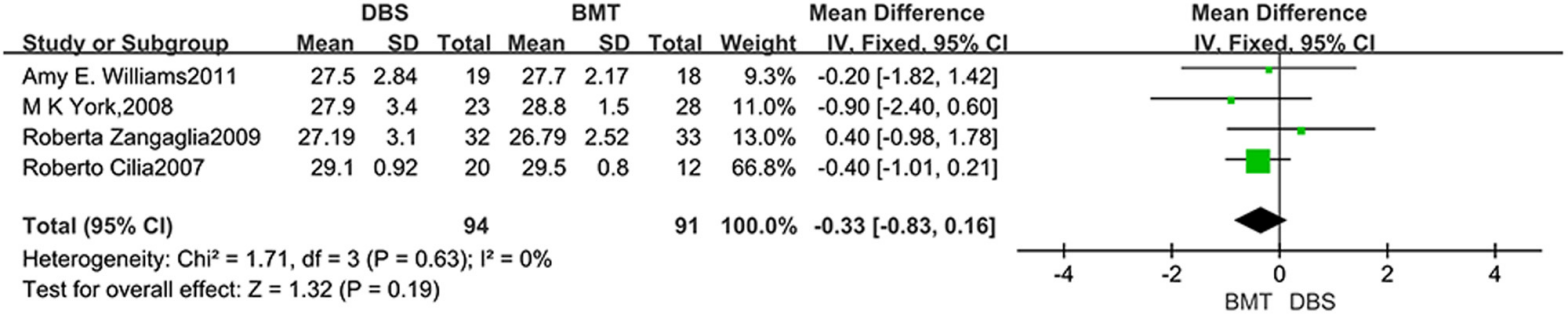
Forest plot of the relationship between DBS and the outcomes of MMSE.

**Figure 3:**
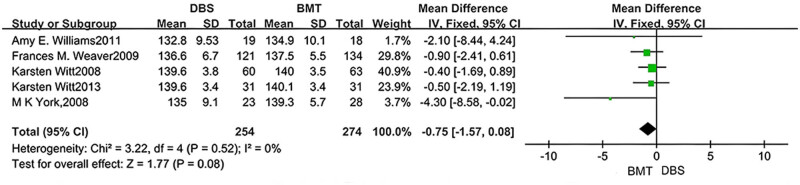
Forest plot of the relationship between DBS and the outcomes of MDRS.

The MMSE was analyzed in four studies. Statistical results showed no significant difference in MMSE results between DBS and BMT groups (MD = −0.33, 95% CI: −0.83 to 0.16, *P* = 0.19, *I* ^2^ = 0%), as shown in Figure 2. The MDRS was analyzed in five studies. The statistical results showed that there was also no significant difference in the MDRS results between the DBS and BMT groups (MD = −0.75, 95% CI: −1.57 to 0.08, *P* = 0.08, *I* ^2^ = 0%), as shown in Figure 3.

### 3.5 Summary of all analyses by domain

In terms of memory functioning, Rey Auditory Verbal Learning Test (RAVLT) learning (*P* = 0.39), RAVLT delay recall (*P* = 0.90), Brief Visuospatial Memory (BVMT) learning scores (*P* = 0.17), and BVMT delay recall scores (*P* = 0.06) did not differ between the two groups. In terms of executive functioning, Trail B (*P* = 0.75), Stroop-color and word scores (*P* = 0.10) and Stroop-color scores (*P* = 0.07) were not significantly different between either group, as shown in Table 2.

**Table 2:** Summary of all the meta-analysis results, grouped by domains

Domain and comparison	Number of studies	MD	95% CI	*P*-value	*I* ^2^ (%)	Effect model	Supplementary information
**General ability**							
MMSE	4	−0.33	−0.83 to 0.16	0.19	0	Fixed	MD < 0, favors BMT
MDRS	5	−0.75	−1.57 to 0.08	0.08	0	Fixed	MD < 0, favors BMT
**Memory**							
RAVLT learning	3	−2.49	−8.19 to 3.22	0.39	73	Random	MD < 0, favors BMT
RAVLT delay recall	3	−0.11	−1.82 to 1.59	0.90	60	Random	MD < 0, favors BMT
BVMT learning	3	−2.86	−6.90 to 1.19	0.17	67	Random	MD < 0, favors BMT
BVMT delay recall	3	−2.19	−4.43 to 0.05	0.06	60	Random	MD < 0, favors BMT
**Information processing**							
Trail A	4	2.63	−7.33 to 12.58	0.60	69	Random	MD > 0, favors BMT
Digit span	6	−0.47	−1.25 to 0.31	0.24	56	Random	MD < 0, favors BMT
Digit span-backward	3	−0.17	−0.68 to 0.34	0.51	0	Fixed	MD < 0, favors BMT
Stroop-word	6	−1.95	−4.93 to 1.04	0.20	65	Random	MD < 0, favors BMT
**Language**							
Letter fluency	3	−6.11	−12.47 to 0.25	0.06	72	Random	MD < 0, favors BMT
Phonemic fluency	7	−3.17	−6.04 to −0.29)	0.03	73	Random	MD < 0, favors BMT
Category fluency	5	−1.61	−3.41 to 0.19	0.08	31	Fixed	MD < 0, favors BMT
Semantic fluency	5	−1.10	−2.43 to 0.24	0.11	0	Fixed	MD < 0, favors BMT
Boston naming	6	−0.46	−1.19 to 0.27	0.22	31	Fixed	MD < 0, favors BMT
**Executive function**							
Trail B	6	6.85	−35.98 to 49.69	0.75	88	Random	MD > 0, favors BMT
Stroop-color	4	−1.33	−2.77 to 0.12	0.07	31	Fixed	MD < 0, favors BMT
Stroop-color and word	6	−11.41	−3.08 to 0.27	0.10	0	Fixed	MD < 0, favors BMT
**Visuospatial**							
JLO	3	−0.23	−1.33 to 0.88	0.69	0	Fixed	MD < 0, favors BMT
**Others**							
PDQ-39 cognition	4	−5.06	−13.5 to 3.39	0.24	64	Random	MD < 0, favors DBS
PDQ-39 communication	4	0.57	−3.48 to 4.62	0.76	0	Fixed	MD > 0, favors BMT

MD, mean difference; 95% CI, 95% confidence intervals; MMSE, Mini-MENTAL State Examination; MDRS, Mattis Dementia Rating Scale; RAVLT, Rey Auditory Verbal Learning Test; BVMT, Brief Visuospatial Memory; JLO, judgment of line orientation; PDQ-39, Parkinson’s disease questionnaire-39; BMT, best medical therapy; DBS, deep brain stimulation.

**Figure 4:**
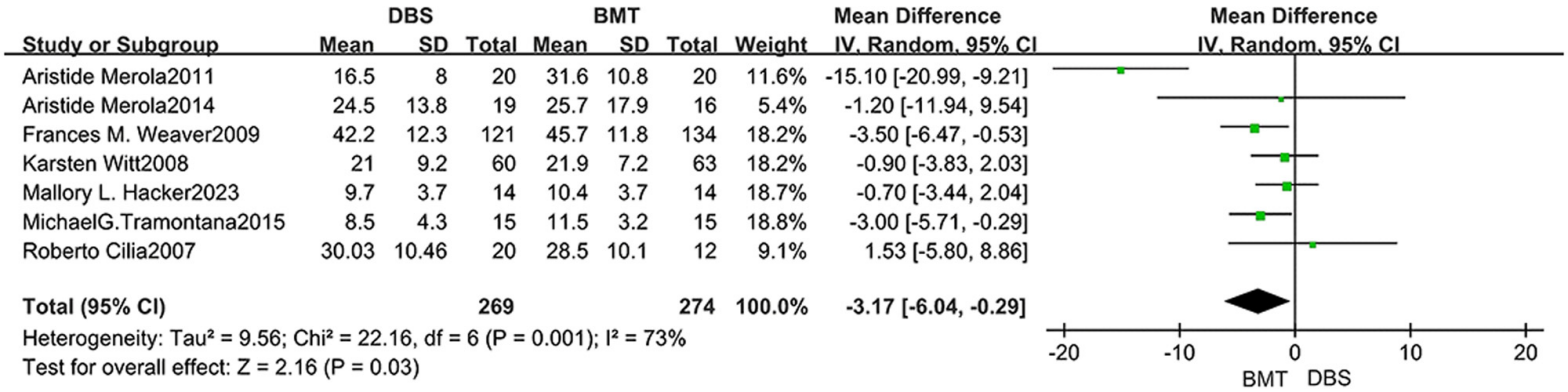
Forest plot of the relationship between DBS and the outcomes of phonemic fluency.

In language, seven studies analyzed phonemic fluency, notably the scores in the DBS group were significantly lower than those in the BMT group (MD = −3.17, 95% CI: −6.04 to −0.29), *P* = 0.03, *I* ^2^ = 73%). The statistical results tended to favor the BMT group, as shown in Figure 4. The scores of semantic fluency (*P* = 0.11), Boston naming (*P* = 0.22), letter fluency (*P* = 0.06), and category fluency (*P* = 0.08) did not differ between the two groups, as shown in Table 2.

In terms of information processing, scores on the Trail A (*P* = 0.60), Digit span (*P* = 0.24), Digit span-backward (*P* = 0.51), and Stroop-word (*P* = 0.20) did not differ significantly between the two groups. In terms of visuospatial, judgment of line orientation (JLO) (*P* = 0.69) scores did not differ significantly between the two groups. In addition to the above scales, the Parkinson’s disease questionnaire-39 (PDQ-39) is also an important tool for evaluating cognitive function. The scores of PDQ-39 cognition (*P* = 0.24) and PDQ-39 communication (*P* = 0.76) did not differ significantly between the two groups, as shown in Table 2.

## 4 Discussion

An increasing number of studies have focused on the therapeutic efficacy and safety of DBS in PD patients [28,29]. There have also been a number of animal studies attempting to find the cause of the improvement in cognitive function in animals by brain stimulation [30,31]. However, whether DBS impairs cognitive function while improving the motor function remains controversial [32,33]. Based on this, this meta-analysis aims to explore the effects of DBS on cognitive function in PD patients, compared to BMT. The results of this study showed that, overall, DBS poses a potential risk to patients’ cognitive functioning, particularly in the phonemic fluency scale, which represents language ability. Also, it showed no possible advantage in the MMSE and MDRS scales, which represent general ability. The BVMT scale (represents memory ability), Stroop scale (represents executive ability), letter fluency scale, and category fluency scale (represents language ability) showed that patients undergoing DBS would have more potential cognitive harms than BMT, although the differences did not reach statistical significance.

The cerebral cortex is the main executive area of cognitive function, and damage or lesions in different areas can lead to different cognitive problems. DBS is a treatment that regulates abnormal neural electrical activity by sending electrical pulses to the associated neural nuclei that control movement through electrodes implanted in the brain, i.e., it can excite abnormal neural nuclei to compensate for the problems caused by neuronal degeneration and loss [34]. However, neural nuclei in a given cortex will inevitably be affected during the stimulation. The prefrontal cortex is closely related to executive function [35]. Damage to the prefrontal cortex will lead to executive dysfunction, mainly manifested as inattention, decreased planning, and decision-making ability. The temporal cortex is closely related to memory function [36]. Damage to the temporal lobe, especially in the hippocampus, can lead to a decline in memory function. However, there are also studies showing that stimulation of the CA3 subregion of the rat hippocampus can help improve memory function in rats with post-traumatic stress disorder [37]. Thus, it seems that further triage of targets and exploration of new targets for DBS targets is worth continuing research.

Studies have shown that after DBS, the functional connections between the bilateral sensorimotor cortex, the right occipital lobe, and the putamen are significantly reduced. In this study, some scales showed potential negative effects of DBS on executive and partial memory functions. The possible reasons include the following: first, DBS can bring about disturbances in neural networks. In a mouse study, van den Boom et al. found that DBS reduces hyperconnectivity between the prefrontal cortex and the striatum, and that this altered connectivity negatively affects memory-related neural networks [38]. Second, DBS may affect the metabolism and release of neurotransmitters. It has been found that high-frequency stimulation leads to depletion or altered release kinetics of presynaptic neurotransmitter vesicles, which further affects neuronal firing patterns and leads to a decline in memory and executive function [39]. Third, DBS may affect sleep architecture and memory consolidation processes during sleep. Hong et al. verified that DBS interfered with prefrontal cortex activity during rapid eye movement sleep by optogenetic means, directly affecting executive function and impairing memory consolidation [40]. In addition, the results of the study by Weinkle et al. were similar to ours [41]. By analyzing the preoperative and ≥6 month postoperative neuropsychological assessment scores of 43 PD patients, they found that cognitive measures such as executive function and memory showed decline after DBS.

Language activity has an extremely complex brain mechanism, which is closely related to different parts of the brain, such as Wernicke’s area, Broca’s area, and angular gyrus [42]. This study included a variety of scales to measure language function. The results of our scale show that there is impairment of language function by DBS. However, it has also been found that DBS improves language function [43]. The reason for the results may be that the scale measuring language function is difficult to operate and depends on the subjective judgment of the operator, which is prone to deviation due to the different experience and observation angle of the evaluator [44,45]. In addition, because of the large number of blocks in the cortex that dominate language, the functional blocks that different scales focus on assessing may be slightly different. For example, letter fluency mainly relies on neural pathways such as the left dorsal prefrontal gyrus and the superior longitudinal fasciculus [46]. This scale requires patients to say as many words beginning with a certain letter as possible in a given period of time, a process that requires the participation of executive functions and language retrieval abilities. Similarly, the completion of semantic fluency also depends on the neural network connection between the prefrontal cortex and the temporal lobe [47]. This is consistent with the results of the present study that DBS can lead to a decline in executive and memory functions.

Whelan et al. found a significant advantage for DBS in enhancing language functioning through the phonemic fluency scale and bilateral STN-DBS may help improve proficiency in the basal-upper cortical language circuit over time [43]. In addition to the above reasons, it should not be ignored that the improvement of motor function in PD patients will further reduce the impairment of verbal fluency [45]. For example, a reduction in laryngeal muscle tremor may improve articulation and fluency. However, the results of the phonemic fluency scale in this study contradicted it. This may be because while DBS modulates motor functions, its targets are also adjacent to these linguistic networks of the left prefrontal–thalamic pathway. Stimulation may indirectly interfere with the process of speech retrieval [48]. Moreover, the frontal-striatal pathway, on which speech fluency is dependent, is sensitive to stimulation. And high-frequency stimulation may inhibit nonmotor networks [49].

Luo et al.’s study obtained fMRI and relatively complete cognitive scale data from 37 PD patients before and after DBS surgery. They found that the scores of general function, information processing ability, and visual space decreased in a short time after DBS surgery, which they thought might be related to the microdamage effect of surgery [50]. However, in the long-term outcome, these functions tended to improve. Our results found no significant differences in general functioning, information processing ability, and visuospatial functioning between the two groups, which may be related to postoperative time according to Luo et al.

Overall, there are some side effects of DBS on patients’ cognitive functions, especially in language functions, where DBS has a significant impairing effect. A number of studies have found that after DBS, patients are prone to mental problems, such as high mood or anxiety and depression [51,52]. In view of this, the use of DBS needs to be emphasized on its side effects and strictly regulated according to the guidelines.

As a conventional type of treatment, DBS has been proved to be effective in the improvement of movement disorders. The main advantage of this study is to explore the effect of DBS on the cognitive function of PD patients, which is informative in the exploration of its safety. Also, this study includes studies from 2002 to 2023, and the results are more universal. However, there are some limitations in this study. First, due to the different types of studies included, as well as the presence of racial differences, different severity of illness, and different drug treatment doses among the subjects included, which inevitably leads to heterogeneity. For example, the scale like phonemic fluency, which is an observational scale, is unavoidable with variations in assessment across studies. Second, subgroup analyses of follow-up time are in need, but additional subgroup analyses were not possible owing to the limited number of included studies. Finally, there were limited RCTs in the included studies, lowering the level of evidence.

## 5 Conclusion

This meta-analysis found that DBS may have potential adverse effects on memory and executive function in the treatment of PD, and a significant detrimental effect on language function. However, larger multicenter RCTs are needed to explore these results.

## Abbreviations

BMT: best medical therapy
BVMT: Brief Visuospatial Memory
DBS: deep brain stimulation
GPi: globus pallidus
JLO: judgment of line orientation
MD: mean difference
MDRS: Mattis Dementia Rating Scale
MMSE: Mini-MENTAL State Examination
PD: Parkinson’s disease
PDQ-39: Parkinson’s disease questionnaire-39
RAVLT: Rey Auditory Verbal Learning Test
RCT: randomized controlled trial
STN: stimulation of the subthalamic nucleus
VIM: ventralis intermedius
95% CI: 95% confidence intervals

## Supplementary Material

Supplementary material

## References

[1] Hayes MT. Parkinson’s disease and parkinsonism. Am J Med. 2019;132(7):802–7. 10.1016/j.amjmed.2019.03.001 30890425

[2] Elsworth JD. Parkinson’s disease treatment: past, present, and future. J Neural Transm. 2020;127(5):785–91. 10.1007/s00702-020-02167-1 PMC8330829 32172471

[3] Beudel M, Brown P. Adaptive deep brain stimulation in Parkinson’s disease. Parkinsonism Relat Disord. 2016;22(Suppl 1):S123–6. 10.1016/j.parkreldis.2015.09.028 PMC4671979 26411502

[4] Marsili L, Bologna M, Miyasaki JM, Colosimo C. Parkinson’s disease advanced therapies – a systematic review: more unanswered questions than guidance. Parkinsonism Relat Disord. 2021;83:132–9. 10.1016/j.parkreldis.2020.10.042 33158747

[5] Limousin P, Foltynie T. Long-term outcomes of deep brain stimulation in Parkinson disease. Nat Rev Neurol. 2019;15(4):234–42. 10.1038/s41582-019-0145-9 30778210

[6] Hong J, Xie H, Chen Y, Liu D, Wang T, Xiong K, et al. Effects of STN-DBS on cognition and mood in young-onset Parkinson’s disease: a two-year follow-up. Front Aging Neurosci. 2023;15:1177889. 10.3389/fnagi.2023.1177889 PMC10824910 38292047

[7] Acera M, Molano A, Tijero B, Bilbao G, Lambarri I, Villoria R, et al. Long-term impact of subthalamic stimulation on cognitive function in patients with advanced Parkinson’s disease. Neurologia. 2019;34(9):573–81. 10.1016/j.nrl.2017.05.009 28712841

[8] Hitti FL, Ramayya AG, McShane BJ, Yang AI, Vaughan KA, Baltuch GH. Long-term outcomes following deep brain stimulation for Parkinson’s disease. J Neurosurg. 2020;132(1):205–10. 10.3171/2018.8.JNS182081 30660117

[9] Yu P, Wang Y, Su F, Chen Y. Comparing [18F]FET PET and [18F]FDOPA PET for glioma recurrence diagnosis: a systematic review and meta-analysis. Front Oncol. 2023;13:1346951. 10.3389/fonc.2023.1346951 PMC10805829 38269019

[10] Just H, Ostergaard K. Health-related quality of life in patients with advanced Parkinson’s disease treated with deep brain stimulation of the subthalamic nuclei. Mov Disord. 2002;17(3):539–45. 10.1002/mds.10111 12112204

[11] Cilia R, Siri C, Marotta G, De Gaspari D, Landi A, Mariani CB, et al. Brain networks underlining verbal fluency decline during STN-DBS in Parkinson’s disease: an ECD-SPECT study. Parkinsonism Relat Disord. 2007;13(5):290–4. 10.1016/j.parkreldis.2006.11.011 17292655

[12] Merola A, Zibetti M, Angrisano S, Rizzi L, Lanotte M, Lopiano L. Comparison of subthalamic nucleus deep brain stimulation and Duodopa in the treatment of advanced Parkinson’s disease. Mov Disord. 2011;26(4):664–70. 10.1002/mds.23524 21469197

[13] Mikos A, Zahodne L, Okun MS, Foote K, Bowers D. Cognitive declines after unilateral deep brain stimulation surgery in Parkinson’s disease: a controlled study using reliable change, part II. Clin Neuropsychol. 2010;24(2):235–45. 10.1080/13854040903277297 PMC3045858 19953428

[14] Smeding HM, Speelman JD, Koning-Haanstra M, Schuurman PR, Nijssen P, van Laar T, et al. Neuropsychological effects of bilateral STN stimulation in Parkinson disease: a controlled study. Neurology. 2006;66(12):1830–6. 10.1212/01.wnl.0000234881.77830.66 16801645

[15] Weaver FM, Follett K, Stern M, Hur K, Harris C, Marks Jr WJ, et al. Bilateral deep brain stimulation vs best medical therapy for patients with advanced Parkinson disease: a randomized controlled trial. JAMA. 2009;301(1):63–73. 10.1001/jama.2008.929 PMC2814800 19126811

[16] Williams A, Gill S, Varma T, Jenkinson C, Quinn N, Mitchell R, et al. Deep brain stimulation plus best medical therapy versus best medical therapy alone for advanced Parkinson’s disease (PD SURG trial): a randomised, open-label trial. Lancet Neurol. 2010;9(6):581–91. 10.1016/S1474-4422(10)70093-4 PMC2874872 20434403

[17] Witt K, Daniels C, Reiff J, Krack P, Volkmann J, Pinsker MO, et al. Neuropsychological and psychiatric changes after deep brain stimulation for Parkinson’s disease: a randomised, multicentre study. Lancet Neurol. 2008;7(7):605–14. 10.1016/S1474-4422(08)70114-5 18538636

[18] York MK, Dulay M, Macias A, Levin HS, Grossman R, Simpson R, et al. Cognitive declines following bilateral subthalamic nucleus deep brain stimulation for the treatment of Parkinson’s disease. J Neurol Neurosurg Psychiatry. 2008;79(7):789–95. 10.1136/jnnp.2007.118786 17965146

[19] Zahodne LB, Okun MS, Foote KD, Fernandez HH, Rodriguez RL, Kirsch-Darrow L, et al. Cognitive declines one year after unilateral deep brain stimulation surgery in Parkinson’s disease: a controlled study using reliable change. Clin Neuropsychol. 2009;23(3):385–405. 10.1080/13854040802360582 PMC3045862 18821180

[20] Zangaglia R, Pacchetti C, Pasotti C, Mancini F, Servello D, Sinforiani E, et al. Deep brain stimulation and cognitive functions in Parkinson’s disease: a three-year controlled study. Mov Disord. 2009;24(11):1621–8. 10.1002/mds.22603 19514093

[21] Blomstedt P, Stenmark Persson R, Hariz GM, Linder J, Fredricks A, Häggström B, et al. Deep brain stimulation in the caudal zona incerta versus best medical treatment in patients with Parkinson’s disease: a randomised blinded evaluation. J Neurol Neurosurg Psychiatry. 2018;89(7):710–6. 10.1136/jnnp-2017-317219 PMC6031280 29386253

[22] Charles D, Konrad PE, Neimat JS, Molinari AL, Tramontana MG, Finder SG, et al. Subthalamic nucleus deep brain stimulation in early stage Parkinson’s disease. Parkinsonism Relat Disord. 2014;20(7):731–7. 10.1016/j.parkreldis.2014.03.019 PMC4103427 24768120

[23] Hacker ML, Tramontana MG, Pazira K, Meystedt JC, Turchan M, Harper KA, et al. Long-term neuropsychological outcomes of deep brain stimulation in early-stage Parkinson’s disease. Parkinsonism Relat Disord. 2023;113:105479. 10.1016/j.parkreldis.2023.105479 PMC11232874 37380539

[24] Merola A, Rizzi L, Zibetti M, Artusi CA, Montanaro E, Angrisano S, et al. Medical therapy and subthalamic deep brain stimulation in advanced Parkinson’s disease: a different long-term outcome? J Neurol Neurosurg Psychiatry. 2014;85(5):552–9. 10.1136/jnnp-2013-305271 23847290

[25] Tramontana MG, Molinari AL, Konrad PE, Davis TL, Wylie SA, Neimat JS, et al. Neuropsychological effects of deep brain stimulation in subjects with early stage Parkinson’s disease in a randomized clinical trial. J Parkinsons Dis. 2015;5(1):151–63. 10.3233/JPD-140448 25613351

[26] Williams AE, Arzola GM, Strutt AM, Simpson R, Jankovic J, York MK. Cognitive outcome and reliable change indices two years following bilateral subthalamic nucleus deep brain stimulation. Parkinsonism Relat Disord. 2011;17(5):321–7. 10.1016/j.parkreldis.2011.01.011 PMC3109216 21316292

[27] Witt K, Granert O, Daniels C, Volkmann J, Falk D, van Eimeren T, et al. Relation of lead trajectory and electrode position to neuropsychological outcomes of subthalamic neurostimulation in Parkinson’s disease: results from a randomized trial. Brain. 2013;136(Pt 7):2109–19. 10.1093/brain/awt151 23801735

[28] Vijiaratnam N, Simuni T, Bandmann O, Morris HR, Foltynie T. Progress towards therapies for disease modification in Parkinson’s disease. Lancet Neurol. 2021;20(7):559–72. 10.1016/S1474-4422(21)00061-2 34146514

[29] Dharnipragada R, Denduluri LS, Naik A, Bertogliat M, Awad M, Ikramuddin S, et al. Frequency settings of subthalamic nucleus DBS for Parkinson’s disease: a systematic review and network meta-analysis. Parkinsonism Relat Disord. 2023;116:105809. 10.1016/j.parkreldis.2023.105809 37604755

[30] Jin K, Chen B, Han S, Dong J, Cheng S, Qin B, et al. Repetitive transcranial magnetic stimulation (rTMS) improves cognitive impairment and intestinal microecological dysfunction induced by high-fat diet in rats. Research. 2024;7:0384. 10.34133/research.0384 PMC11140411 38826566

[31] Wang SS, Mao XF, Cai ZS, Lin W, Liu XX, Luo B, et al. Distinct olfactory bulb-cortex neural circuits coordinate cognitive function in parkinson’s disease. Research. 2024;7:0484. 10.34133/research.0484 PMC11445789 39359881

[32] Zheng W, Lv G, Lu Y, Liu J, Hao Q, Ding H, et al. Bilateral pallidal deep brain stimulation in meige syndrome: effects on motor function, neuropsychological status, and mood. Neurosurgery. 2023;92(5):1073–9. 10.1227/neu.0000000000002335 36728352

[33] Pal GD, Corcos DM, Metman LV, Israel Z, Bergman H, Arkadir D. Cognitive effects of subthalamic nucleus deep brain stimulation in Parkinson’s disease with GBA1 pathogenic variants. Mov Disord. 2023;38(12):2155–62. 10.1002/mds.29647 PMC10990226 37916476

[34] Whitestone J, Salih A, Goswami T. Investigation of a deep brain stimulator (DBS) system. Bioengineering. 2023;10(10):1160. 10.3390/bioengineering10101160 PMC10604713 37892890

[35] Damrongthai C, Kuwamizu R, Suwabe K, Ochi G, Yamazaki Y, Fukuie T, et al. Benefit of human moderate running boosting mood and executive function coinciding with bilateral prefrontal activation. Sci Rep. 2021;11(1):22657. 10.1038/s41598-021-01654-z PMC8608901 34811374

[36] Bachmann D, Buchmann A, Studer S, Saake A, Rauen K, Zuber I, et al. Age-, sex-, and pathology-related variability in brain structure and cognition. Transl Psychiatry. 2023;13(1):278. 10.1038/s41398-023-02572-6 PMC10423720 37574523

[37] Yu Y, Wu K, Yang X, Long J, Chang C. Terahertz photons improve cognitive functions in posttraumatic stress disorder. Research. 2023;6:0278. 10.34133/research.0278 PMC10726292 38111677

[38] van den Boom BJG, Elhazaz-Fernandez A, Rasmussen PA, van Beest EH, Parthasarathy A, Denys D, et al. Unraveling the mechanisms of deep-brain stimulation of the internal capsule in a mouse model. Nat Commun. 2023;14(1):5385. 10.1038/s41467-023-41026-x PMC10477328 37666830

[39] Neumann WJ, Steiner LA, Milosevic L. Neurophysiological mechanisms of deep brain stimulation across spatiotemporal resolutions. Brain. 2023;146(11):4456–68. 10.1093/brain/awad239 PMC10629774 37450573

[40] Hong J, Choi K, Fuccillo MV, Chung S, Weber F. Infralimbic activity during REM sleep facilitates fear extinction memory. Curr Biol. 2024;34(10):2247–55.e5. 10.1016/j.cub.2024.04.018 PMC11111341 38714199

[41] Weinkle LJ, Hoyt B, Thompson JA, Sillau S, Tanabe J, Honce J, et al. Association of MRI measurements with cognitive outcomes after STN-DBS in Parkinson’s disease. Mov Disord Clin Pract. 2018;5(4):417–26. 10.1002/mdc3.12643 PMC6174386 30363383

[42] Tremblay P, Dick AS. Broca and Wernicke are dead, or moving past the classic model of language neurobiology. Brain Lang. 2016;162:60–71. 10.1016/j.bandl.2016.08.004 27584714

[43] Whelan BM, Murdoch BE, Theodoros DG, Silburn P, Hall B. Beyond verbal fluency: investigating the long-term effects of bilateral subthalamic (STN) deep brain stimulation (DBS) on language function in two cases. Neurocase. 2005;11(2):93–102. 10.1080/13554790590925501 16036464

[44] Chiu SY, Tsuboi T, Hegland KW, Herndon NE, Shukla AW, Patterson A, et al. Dysarthria and speech intelligibility following Parkinson’s disease globus pallidus internus deep brain stimulation. J Parkinsons Dis. 2020;10(4):1493–502. 10.3233/JPD-202246 32955467

[45] Pedrosa DJ, Auth M, Pauls KA, Runge M, Maarouf M, Fink GR, et al. Verbal fluency in essential tremor patients: the effects of deep brain stimulation. Brain Stimul. 2014;7(3):359–64. 10.1016/j.brs.2014.02.012 24661791

[46] Tabari F, Berger JI, Flouty O, Copeland B, Greenlee JD, Johari K. Speech, voice, and language outcomes following deep brain stimulation: a systematic review. PLoS One. 2024;19(5):e0302739. 10.1371/journal.pone.0302739 PMC11086900 38728329

[47] Tang V, Zhu CX, Chan D, Lau C, Chan A, Mok V, et al. Evidence of improved immediate verbal memory and diminished category fluency following STN-DBS in Chinese-Cantonese patients with idiopathic Parkinson’s disease. Neurol Sci. 2015;36(8):1371–7. 10.1007/s10072-015-2117-1 25708249

[48] Hariz M, Blomstedt P. Deep brain stimulation for Parkinson’s disease. J Intern Med. 2022;292(5):764–78. 10.1111/joim.13541 PMC9796446 35798568

[49] Witt K, Daniels C, Volkmann J. Factors associated with neuropsychiatric side effects after STN-DBS in Parkinson’s disease. Parkinsonism Relat Disord. 2012;18(Suppl 1):S168–70. 10.1016/S1353-8020(11)70052-9 22166423

[50] Luo B, Zou Y, Yan J, Sun J, Wei X, Chang L, et al. Altered cognitive networks connectivity in parkinson’s disease during the microlesion period after deep brain stimulation. CNS Neurosci Ther. 2024;30(12):e70184. 10.1111/cns.70184 PMC11669568 39722165

[51] Mosley PE, Akram H. Neuropsychiatric effects of subthalamic deep brain stimulation. Handb Clin Neurol. 2021;180:417–31. 10.1016/B978-0-12-820107-7.00026-4 34225945

[52] Olaciregui Dague K, Witt JA, von Wrede R, Helmstaedter C, Surges R. DBS of the ANT for refractory epilepsy: a single center experience of seizure reduction, side effects and neuropsychological outcomes. Front Neurol. 2023;14:1106511. 10.3389/fneur.2023.1106511 PMC10033684 36970547

