# Supplementary material for: A meta-analysis of the effects of DBS on cognitive function in patients with advanced PD
Source: Open Med (Wars). 2025 Oct 9;20(1):20251292. doi: 10.1515/med-2025-1292 (PMC12514775; doi:10.1515/med-2025-1292)
Supplement: Supplementary material [file med-2025-1292-sm.pdf]

Supplementary material

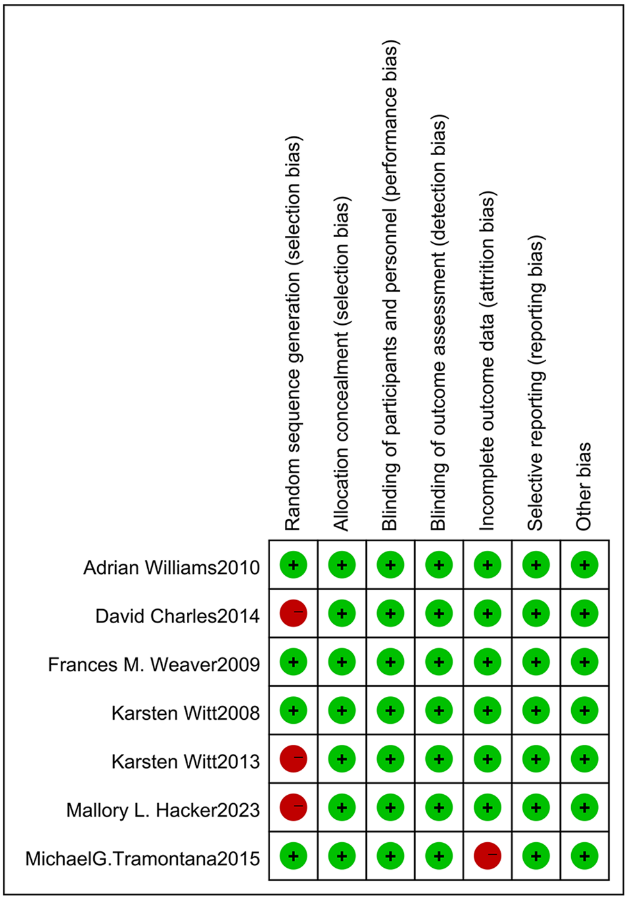

Figure S1: Summary of the risk of bias based on the evaluation domains listed in the Cochrane Collaboration Risk of Bias Tool.

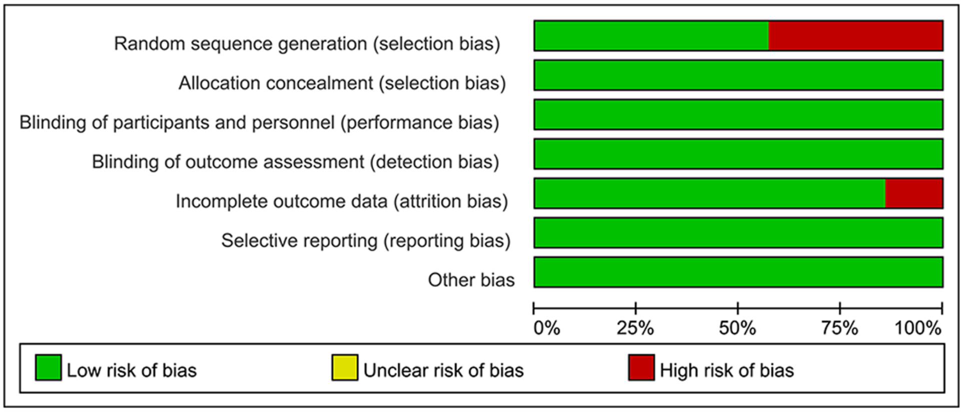

Figure S2: Proportion of risk of bias based on the evaluation domains listed in the Cochrane Collaboration Risk of Bias Tool.

Table S1: Characteristics of all the studies included in the meta-analysis

Author	Year	Registered ID (randomization)	Median age (years)		Duration of disease (years)		Proportion of males (%)		Medical therapy (Levodopa equivalents mg/d)	
			Experimental	Control	Experimental	Control	Experimental	Control	Experimental	Control
Helle just	2002	—	51.8–70.9	53.8–69.2	7.0–25.0	10.0–27.0	8 (72.0%)	7 (54.0%)	—	—
H.M.M. Smeding	2006	—	57.9 ± 8.1	63.0 ± 9.1	13.7 ± 6.1	10.4 ± 4.6	58 (58.6%)	21 (58.3%)	—	—
Roberto cilia	2007	—	59.2 ± 7.4	61.3 ± 5.3	13.27 ± 3.1	15.27 ± 4.4	14 (70.0%)	10 (83.3%)	—	—
Karsten witt	2008	NCT00196911	60.2 ± 7.9	59.4 ± 7.5	13.8 ± 6.3	14.0 ± 6.1	36 (60.0%)	41 (65.1%)	1203 ± 535	1142 ± 463
M K York	2008	—	59.5 ± 11.8	66.7 ± 8.7	12.0 ± 5.5	4.7 ± 4.4	13 (57.0%)	20 (74.0%)	1009 ± 445	647 ± 420
Frances M. weaver	2009	NCT00056563	62.4 ± 8.8	62.3 ± 9.0	—	—	98 (81.0%)	110 (82.1%)	1281 ± 521	1289 ± 546
Laura B. Zahodne	2009	—	61.4 ± 5.0	64.6 ± 6.6	—	—	18 (81.8%)	12 (63.2%)	1207 ± 305	1190 ± 718
Roberta zangaglia	2009	—	58.8 ± 7.7	62.5 ± 6.8	11.8 ± 5.1	9.9 ± 4.9	18 (56.3%)	20 (60.6%)	617 ± 304	648 ± 244
Ania mikos	2010	—	61.7 ± 4.9	64.7 ± 6.6	11.5 ± 5.3	6.3 ± 5.7	20 (83.3%)	12 (63.2%)	—	—
Adrian williams	2010	ISRCTN34111222	59.0 (37.0–79.0)	59.0 (36.0–75.0)	11.5 (2.0–32.2)	11.2 (1.0–30.0)	125 (68.0%)	135 (74.0%)	897 ± 568	1347 ± 585
Aristide merola	2011	—	66.6 ± 2.5	69.0 ± 5.9	16.4 ± 4.3	13.9 ± 4.5	16 (80.0%)	13 (65.0%)	982 ± 315	907 ± 249
Amy E. williams	2011	—	62.1 ± 10.3	66.6 ± 9.0	10.1 ± 6.2	7.5 ± 4.2	10 (53.0%)	15 (83.0%)	468 ± 293	1018 ± 411
Karsten witt	2013	NCT00196911	59.8 ± 7.5	58.9 ± 9.6	13.3 ± 5.5	13.1 ± 5.3	17 (54.8%)	17 (54.8%)	1244 ± 527	1204 ± 522
Aristide merola	2014	—	60.1 ± 5.6	60.9 ± 5.8	12.9 ± 2.2	11.1 ± 2.9	—	—	1120 ± 329	1253 ± 430
David charles	2014	NCT00282152	60.0 ± 6.8	60.0 ± 7.0	2.2 ± 1.4	2.1 ± 1.1	14 (93.3%)	13 (86.7%)	417 ± 307	494 ± 209
MichaelG.tramontana	2015	NCT00282152	60.0 ± 6.8	60.0 ± 7.0	2.2 ± 1.4	2.1 ± 1.1	14 (93.3%)	13 (86.7%)	417 ± 307	494 ± 209
Patric blomstedt	2018	—	57.0 ± 11.4	60.9 ± 9.2	6.4 ± 3	10.3 ± 5.6	7 (77.8%)	8 (80.0%)	1376 ± 883	1043 ± 516
Mallory L. hacker	2023	NCT00282152	61.4 ± 6.4	60.7 ± 6.6	—	—	12 (85.7%)	13 (92.6%)	409 ± 316	491 ± 216

Table S2: Quality assessment of cohort studies included

Author, year	Selection (Out of 4)				Comparability (Out of 2)	Outcomes (Out of 3)			Total (Out of 9)
	Representativeness of exposed cohort	Selection of non exposed cohort	Ascertainment of exposure	Outcome not present at the start of the study		Assessment of outcomes	Length of follow-up	Adequacy of follow up of cohorts	
Helle Just. 2002	1	1	1	1	1	1	1	1	8
H.M.M. Smeding. 2006	1	1	1	1	2	1	0	1	8
Roberto cilia. 2007	1	1	1	1	1	1	0	1	7
M K york. 2008	1	1	1	1	1	1	1	1	8
Laura B. zahodne. 2009	1	1	1	1	2	1	1	1	9
Roberta zangaglia. 2009	1	1	1	1	1	0	0	1	6
Ania mikos. 2010	1	1	1	1	1	1	0	0	6
Aristide merola. 2011	1	1	1	1	1	1	1	0	7
Amy E. williams. 2011	1	1	1	1	1	1	1	0	7
Aristide merola. 2014	1	1	1	1	1	1	1	0	7
Patric blomstedt. 2018	1	1	1	1	1	1	0	1	7
